# Measles Outbreak Among Members of a Religious Community — Brooklyn, New York, March–June 2013

**Published:** 2013-09-13

**Authors:** Robert J. Arciuolo, Tamara R. Brantley, Mekete M. Asfaw, Rachel R. Jablonski, Jie Fu, Francesca R. Giancotti, Jennifer B. Rosen, Jane R. Zucker

**Affiliations:** New York City Dept of Health and Mental Hygiene; Immunization Svcs Div, National Center for Immunization and Respiratory Diseases, CDC

On March 13, 2013, an intentionally unvaccinated adolescent aged 17 years returned to New York City from London, United Kingdom, while infectious with measles. This importation led to the largest outbreak of measles in the United States since 1996 (1).

Investigation of suspected cases included patient interviews, medical record reviews, and ascertainment of immunization records. Testing for measles immunoglobulin G (IgG) and immunoglobulin M (IgM) and testing for measles virus RNA by reverse-transcription polymerase chain reaction (RT-PCR) were performed, and measles genotype was determined. Cases were identified in residents of New York City and classified according to the Council of State and Territorial Epidemiologists clinical case definition (2). Exposed contacts were identified, and control measures were implemented.

A total of 58 cases* were identified, including six generations of measles infection in two neighborhoods of the borough of Brooklyn. All cases were in members of the orthodox Jewish community. No case was identified in a person who had documented measles vaccination at the time of exposure; 12 (21%) of the cases were in infants too young (aged <12 months) for routine immunization with measles, mumps, and rubella (MMR) vaccine.

The outbreak was first recognized in Brooklyn’s Borough Park neighborhood, where the median age of 28 infected persons was 10 years (range: 0–32 years), and 79% of cases in persons aged ≥12 months were in three extended families whose members declined use of measles vaccine. The outbreak spread to the Williamsburg neighborhood, where the median age of 30 infected persons was 19 months (range: 0 months–32 years), and the primary reasons for lack of vaccination were refusal (nine, 30%) and delay (eight, 27%). Forty-eight (83%) of all cases were confirmed by positive measles IgM or RT-PCR result and 10 (17%) by epidemiologic linkage (2). Genotype D8 was identified in 17 cases, consistent with known current circulation of this genotype in the United Kingdom. No other genotype was identified among the cases.

In 31% of cases, no medical care for rash illness had been sought and, therefore, the cases had not been reported to the New York City Department of Health and Mental Hygiene (DOHMH) by a medical provider. In 9% of cases, patients saw a medical provider at the time of rash illness but were not reported when the diagnosis of measles was first considered. In 52% of cases, measles was likely acquired from a relative. Complications included pneumonia in one child; two pregnant women required hospitalization, including one who miscarried. The last case onset occurred on June 9, 2013.

Approximately 3,500 contacts were identified in health-care, school, and home settings. Control measures included administration of immune globulin or MMR vaccine post-exposure prophylaxis; home isolation; alerts to medical providers; active recall of children in medical practices who were not up-to-date with measles vaccine; notifications to families, schools and day care providers through letters, flyers, and advertisements in newspapers; immunization audits of schools; and meetings with religious leaders and elected officials. DOHMH recommended that obstetricians in affected communities test for measles immunity during pregnancy and vaccinate women without evidence of measles immunity postpartum. Because infants were affected, vaccination recommendations during the outbreak period were expanded to include MMR vaccine for all children aged 6–11 months in the affected communities, with the second dose of MMR vaccine administered early, as soon as 4 weeks after the first dose of MMR vaccine.

Measles elimination was declared in the United States in 2000. However, importations of measles continue to present risks for outbreaks in the United States. This outbreak was propagated by a few extended families whose members declined MMR vaccine and by children with delays in receiving MMR vaccine in densely populated neighborhoods (3). High vaccination coverage within the Brooklyn orthodox Jewish community likely limited the scope of the outbreak. The insular nature of the affected community and high population-level vaccination coverage outside this community likely prevented further spread of measles.

## Footnotes

* Includes 57 confirmed cases and one case in a newborn who was culture-positive and born to an infected mother but did not have documentation of clinical symptoms.
